# Adaptation and diversity along an altitudinal gradient in Ethiopian barley (*Hordeum vulgare *L.) landraces revealed by molecular analysis

**DOI:** 10.1186/1471-2229-10-121

**Published:** 2010-06-21

**Authors:** Tesema Tanto Hadado, Domenico Rau, Elena Bitocchi, Roberto Papa

**Affiliations:** 1 Dipartimento di Scienze Ambientali e delle Produzioni Vegetali, Università Politecnica delle Marche, Via Brecce Bianche, 60131 Ancona, Italy; 2 Institute of Biodiversity Conservation, P.O. Box 30726, Addis Ababa, Ethiopia; 3 Dipartimento di Scienze Agronomiche e Genetica Vegetale Agraria, Università degli Studi di Sassari, Via E. De Nicola, 07100, Sassari, Italy

## Abstract

**Background:**

Among the cereal crops, barley is the species with the greatest adaptability to a wide range of environments. To determine the level and structure of genetic diversity in barley (*Hordeum vulgare *L.) landraces from the central highlands of Ethiopia, we have examined the molecular variation at seven nuclear microsatellite loci.

**Results:**

A total of 106 landrace populations were sampled in the two growing seasons (*Meher *and *Belg*; the long and short rainy seasons, respectively), across three districts (Ankober, Mojanawadera and Tarmaber), and within each district along an altitudinal gradient (from 1,798 to 3,324 m a.s.l). Overall, although significant, the divergence (e.g. F_ST_) is very low between seasons and geographical districts, while it is high between different classes of altitude. Selection for adaptation to different altitudes appears to be the main factor that has determined the observed clinal variation, along with population-size effects.

**Conclusions:**

Our data show that barley landraces from Ethiopia are constituted by highly variable local populations (farmer's fields) that have large within-population diversity. These landraces are also shown to be locally adapted, with the major driving force that has shaped their population structure being consistent with selection for adaptation along an altitudinal gradient. Overall, our study highlights the potential of such landraces as a source of useful alleles. Furthermore, these landraces also represent an ideal system to study the processes of adaptation and for the identification of genes and genomic regions that have adaptive roles in crop species.

## Background

Among the cereal crops, barley is the species that presents the highest adaptability to a wide range of environments. It is cultivated from arctic latitudes to tropical areas, and it is grown at the highest altitudes. In Tibet, Nepal, Ethiopia and the Andes, farmers cultivate barley on mountain slopes at altitudes higher than any other cereals [1,2].

Ethiopia is probably the region of barley cultivation that presents the highest variability for climatic and edaphic conditions. It is cultivated from 1,400 m above sea level (a.s.l.) to over 4,000 m a.s.l., and it has adapted to specific sets of agro-ecological and microclimatic regimes throughout the country [3]. Landraces represent over 90% of the barley cultivated in Ethiopia. In contrast to the genetic uniformity of modern cultivars, landraces show variation both between and within populations. This within-population diversity of these barley landraces might allow them to cope with environmental stresses, which is very important for achieving yield stability [4]. For this reason, landraces represent a very interesting model to study the processes of adaptation and for identification of genes and genomic regions that have adaptive roles in a crop species, through association mapping [5-8] and through scanning for signatures of selection at the molecular level [9-13].

Knowledge of the population structure of Ethiopian barley landraces, together with a deeper understanding of the nature and extent of their variation, is an important prerequisite for efficient conservation and use of the existing plant materials. Several studies have reported a high level of genetic diversity in barley populations from Ethiopia, such as those based on morphological traits [14-16], and on biochemical data [17,18]. Moreover, Ethiopian barley is a precious source of genes that control important agronomic traits, such as resistance to disease (e.g. powdery mildew, barley yellow dwarf virus, net blotch, scald and loose smut) and to insect attack [19-25], high lysine, and protein quality and content [26], and malting and brewing quality [27]. Furthermore, the great variability of environmental conditions in Ethiopia that promote adaptive divergence, and the cultivation of barley in two growing seasons per year [16], have probably driven the structure of variation of these landraces.

Numerous studies on Ethiopian barley have detected correlations between altitude and the frequency of morphological [14-16,28], agronomic [24,29] and biochemical [30] traits, including pathogen resistance [20,24,25,29]. Altitudinal gradients provide substantial changes in numerous environmental variables, which also include atmospheric pressure, temperature, clear-sky turbidity, UV-B radiation, and humidity. Furthermore, the combined environmental changes across altitudes can influence various biological processes in plants, and can thus result in adaptive changes and constraints on the genetic diversity of plant populations. Association between environmental variables and allele frequencies can be maintained by a balance between selection and gene flow [31-33]. However, in contrast to latitudinal clines, altitudinal gradients involve dramatic ecological transitions over relatively short linear distances, and so they require especially strong selection to counterbalance the homogenising effects of gene flow [34]. Nevertheless, the interactions between drift and spatially restricted gene flow or historical patterns of colonisation (admixture between previously isolated populations) might also explain clinal variation.

The present study is based on a large collection of Ethiopian barley landraces (3,170 individual plants, for a total of 106 landrace populations) that was established in 2005 in the North Shewa zone (Amhara region, Ethiopia), from farmers who have used their own seed for generations [16]. The collection was obtained through visiting the same three districts (Ankober, Mojanawadera and Tarmaber) in both the *Meher *and *Belg *growing seasons [16] (see [16], for further details). Here, we have analysed the molecular diversity and genetic structure of this collection, using simple sequence repeats (SSRs), while taking into account not only the environmental adaptation (geographical and altitude factors), but also, for the first time, the two growing seasons per year.

## Results

### Level of polymorphism

Seven mapped nuclear SSRs were used to examine the levels and patterns of genetic variation of the barley landraces collected in North Shewa, in the central highlands of Ethiopia [16]. Table 1 provides a summary of the statistics computed considering each of the two seasons (*Belg *and *Meher*), the three districts (Ankober, Mojanawadera, and Tarmaber) and the three altitude classes (<2,300, 2,300-2,800, >2,800 m a.s.l.). The same statistics were also computed for each locus, and are given in Additional file 1. A total of 66 alleles were detected, with the number of alleles per locus (*n_a_*) ranging from four (HVM20 and HVM67) to 23 (Bmac0156), with an average of 9.4 alleles per locus.

**Table 1:** Summary statistics for the seasons, districts and altitude classes, and for the whole sample.

	*S*	***n***_***a***_	***n***_***o***_	***n***_***e***_	*He*	*Ho*	***R***_***S***_	Number of private alleles	Average frequency of private alleles	Number of private alleles (freq. ≥ 0.05)	Average frequency of private alleles (freq. ≥ 0.05)
**Seasons**											
*Belg*	108	60	8.57	4.71	0.63	0.001	8.57	8	0.01	0	-
*Meher*	104	58	8.29	4.54	0.67	0.004	8.28	6	0.02	0	-
**Districts**											
Ankober	72	58	8.29	4.86	0.66	0.006	8.22	9	0.03	2	0.07
Mojanawadera	64	53	7.57	4.67	0.63	0.002	7.57	4	0.03	1	0.05
Tarmaber	76	50	7.14	3.86	0.64	0.000	7.11	2	0.02	0	-
**Altitude classes (m a.s.l.)**											
<2,300	46	39	5.57	3.11	0.65	0.000	5.57	2	0.04	1	0.07
2,300-2,800	54	50	7.14	4.45	0.66	0.000	7.10	3	0.02	0	-
>2,800	112	57	8.14	4.24	0.57	0.009	7.40	12	0.03	3	0.09
**ALL**	**212**	**66**	**9.43**	**4.96**	**0.66**	**0.003**	**9.43**	-	-	-	-

*S*, sample size; *n*_*a*_, number of observed alleles; *n*_*o*_, average number of observed alleles per locus; *n*_*e*_, effective number of alleles per locus; *He*, unbiased expected heterozygosity; *Ho*, observed heterozygosity; *R*_*S*_, allelic richness.

The variation between seasons was not significant across any of the statistics considered (Wilcoxon signed-paired rank test), while the only significant difference among the districts (Wilcoxon signed-paired rank test, after Bonferroni correction, P = 0.03) was for the allelic richness between the Ankober (*R*_*S *_= 8.22) and Tarmaber districts (*R*_*S *_= 7.11; Table 1). For the altitude classes, more marked differences were seen (Table 1). The number of alleles (*n*_*a*_) and the allelic richness (*R*_*S*_) of the low altitude class (<2,300 m a.s.l.: *n*_*a *_= 39 and *R*_*S *_= 5.57) were lower than those of the intermediate altitude class (2,300-2,800 m a.s.l.: *n*_*a *_= 50 and *R*_*S *_= 7.10; difference marginally non-significant, P = 0.06, Wilcoxon signed-paired rank test, after Bonferroni correction) and of the high altitude class (>2800 m a.s.l.: *n*_*a *_= 57, P = 0.06, and *R*_*S *_= 7.40, P = 0.09). The genetic diversity (*He*) of the intermediate altitude class (*He *= 0.65) was significantly higher (Wilcoxon signed-paired rank test, after Bonferroni correction, P = 0.03) than that of the high altitude class (*He *= 0.57).

Considering the pattern of private alleles, the two seasons did not share 14 (21%) out of the 66 SSR alleles, with each of these showing a frequency lower than 5%. The three districts showed a total of 15 private alleles (23%), three of which had a frequency higher than 5%. Finally, compared to seasons and districts, the altitude classes had a higher number of private alleles (17; 26%), four of which (one for <2,300 m a.s.l., and three for >2,800 m a.s.l.) had a frequency higher than 5%. Moreover, the average frequency of private alleles that showed a frequency higher than 5% was 0.00 for seasons, 0.063 for districts, and 0.085 for altitude classes.

The overall level of observed heterozygosity was very low (0.003). Only three individuals, all located at high altitude, showed one or two heterozygous loci. Overall, the average genetic diversity (*He*, [35]) in the Ethiopian barley landraces was 83.5% of the diversity found in the Syrian and Jordanian barley landraces (Table 2). This difference was significant (Wilcoxon signed-paired rank test, P = 0.05). A significant gap (-31.2%) was also seen for the number of alleles, *n*_*a *_(Wilcoxon signed-paired rank test, P = 0.01).

**Table 2:** Diversity of landraces from Ethiopia compared with those from Syria, Jordan and Nepal, and with modern varieties.

	SSR locus name															
			
	HVM20 (ch1H)		Bmac0134 (ch2H)		Bmag0013 (ch3H)		HVM67 (4H)		Bmac0113 (ch5H)		Bmac0040 (ch6H)		Bmac0156 (ch7H)		***Total n***_***a***_	*Mean H*
		
	***n***_***a***_	*H*	***n***_***a***_	*H*	***n***_***a***_	*H*	***n***_***a***_	*H*	***n***_***a***_	*H*	***n***_***a***_	*H*	***n***_***a***_	*H*		
**Ethiopian landraces (209)**	4	0.32	11	0.80	6	0.81	4	0.41	7	0.47	11	0.86	23	0.93	66 (62)^1^	0.66 (0.71)^1^
**Syrian and Jordanian landraces (125)**^**2**^	5	0.56	17	0.90	11	0.80	5	0.65	9	0.80	18	0.88	31	0.91	96 (91)^1^	0.79 (0.82)^1^
**Nepalese landraces (107)**^**3**^	-	-	3	0.44	8	0.26	5	0.30	9	0.83	2	0.02	17	0.87	44	0.50
**Modern varieties (24)**^**3**^	-	-	7	0.79	6	0.81	3	0.50	6	0.79	12	0.94	9	0.84	41	0.64

^1 ^Total number of alleles and average diversity across loci (in parentheses) are calculated excluding the HVM20 locus, to allow direct comparisons with the Nepalese landraces and with the modern varieties.

^2 ^Data from Table 1 of Russel *et al. *(2003).

^3 ^Data from Table 2 of Pandey *et al. *(2006) for the Nepalese landraces, and from the graphical genotypes represented in Figure 2 of Macaulay *et al. *(2001) for the modern varieties. In both of these cases, genetic diversity is calculated as polymorphic information content (PIC), following Weber (1990).

Although it is important to consider the differences in the sample sizes, the Ethiopian barley landraces showed a higher number of alleles (*n*_*a *_= 62, not considering the HVM20 locus) than seen for both the Nepalese barley landraces (*n*_*a *_= 44) [36] and the modern varieties (*n*_*a *_= 41) [37] (Table 2).

The divergence (*F*_ST _and *R*_ST_) estimates between the seasons, districts, and altitude classes are given in Table 3. The genetic differentiation between the two seasons was low for both *F*_ST _(0.02) and *R*_ST _(0.01), even if in contrast to *R*_ST_, the *F*_ST _was significantly different from zero (P < 0.001). A slightly higher level of differentiation was seen for districts (*F*_ST _= 0.02, P < 0.01; *R*_ST _= 0.03, P < 0.05). Among the altitude classes, the levels of differentiation were higher compared to seasons and districts, both for *F*_ST _(0.10, P < 0.001) and *R*_ST _(0.09, P < 0.001). The divergence (*F*_ST _and *R*_ST_) estimates between seasons, districts and altitude classes for each of the SSR loci analysed are given in Additional file 2. The individual SSR loci divergence (*F*_ST_,) between seasons was significant for four loci (Bmag0013, HVM67, Bmac0040, and Bmac0134), even if the values were low, and varied from 0.02 to 0.05 (Additional file 2). The Bmag0013 locus was the only one for which the divergence was higher between seasons (*F*_ST _= 0.05) than among districts or altitude classes (*F*_ST _= 0.01 and 0.03 respectively; Additional file 2). A similar trend was seen for districts, with three low significance values ranking from 0.02 (Bmac0156) to 0.04 (Bmac0134). For the altitude classes, the situation was different; indeed, all of the SSR loci were significantly differentiated among the altitude classes, with values varying from 0.03 (Bmag0013) to 0.28 (HVM67).

**Table 3:** Divergence (*F*_ST _and *R*_ST_) estimates between seasons, districts and altitude classes.

	***F***_**ST**_	***R***_**ST**_
**Seasons**	0.02***	0.01
**Districts**	0.02**	0.03*
**Altitude classes**	0.10***	0.09***

*P < 0.05; **P < 0.01; ***P < 0.001.

Table 4 reports the divergence estimates (*F*_ST_) among the three districts within the same altitude classes (<2,300 m a.s.l.; 2,300-2,800 m a.s.l., and >2,800 m a.s.l.). The differentiation among the low altitude classes of the three districts was higher (*F*_ST _= 0.10, P < 0.01) than that among the three districts within the intermediate (*F*_ST _= 0.04, P > 0.05) and high (*F*_ST _= 0.01, P > 0.05) altitude classes.

**Table 4:** Divergence (*F*_ST_) estimates between the three different districts within the same altitude classes.

<2,300 m a.s.l. classes	***F***_**ST**_
Ankober - Mojanawadera	0.16**
Ankober - Tarmaber	0.10*
Mojanawadera - Tarmaber	0.19*
Mean *F*_ST_	0.15
**Average unweighted *F***_**ST**_	0.10**

**2,300-2,800 m a.s.l. classes**	

Ankober - Mojanawadera	0.08*
Ankober - Tarmaber	0.05 ns
Mojanawadera - Tarmaber	0.07*
Mean *F*_ST_	0.07
**Average unweighted *F***_**ST**_	**0.04 ns**

**>2,800 m a.s.l. classes**	

Ankober - Mojanawadera	0.03*
Ankober - Tarmaber	0.03 ns
Mojanawadera - Tarmaber	0.02 ns
Mean *F*_ST_	0.03
**Average unweighted *F***_**ST**_	**0.01 ns**

ns, not significant

*P < 0.05, **P < 0.01

### Population structure and altitude cline of genetic variation

Recently, various studies (mainly in the field of human genetics [38,39]) have questioned the reliability of the STRUCTURE results in cases of complex genetic structures. In particular, this debate has considered whether Bayesian clustering algorithms are appropriate tools for studying genetic structures in populations with continuous variation of allele frequencies. To address this problem, the population structure analysis of our collection was carried out using two different methods: the first was the Pritchard *et al. *[40] non-spatial Bayesian clustering method implemented in the STRUCTURE software, version 2.1 [41], and the second was based on a Bayesian clustering algorithm that incorporates spatial information when identifying clusters of individuals, and was implemented in the TESS software, version 2.0 [42].

The plot of the average *ln likelihood *values over 100 runs for *K *values ranging from 1 to 10 shows that the *ln likelihood *estimates increase progressively as *K *increases. Thus, we used the Evanno *et al. *[43] method to provide a better estimate of the 'true' number of clusters (*K*) that characterised our sample. The highest Δ*K *value was found at *K *= 2, although high values of Δ*K *were found also for *K *= 3, 4 and 10. To minimise the effects of outlier runs, we also computed the Δ*K *value considering five random re-samplings of 80 of the 100 runs for each *K *value. In all cases, the highest Δ*K *corresponded to *K *= 2. Thus, hereafter, we will refer to cluster S1 and cluster S2 for the *K *= 2 populations identified by STRUCTURE.

The percentages of membership (*q*), or inferred group ancestries, of the individuals in each of the two clusters were computed. One hundred and ninety-seven individuals (93%) had a *q *higher than 0.70, in 192 individuals (91%), *q *was higher than 0.80, and in 183 (86%), it was higher than 0.90. Considering the threshold for membership of *q *≥ 0.70, cluster S1 included 110 individuals, and cluster S2, 87 individuals.

**Figure 1:**
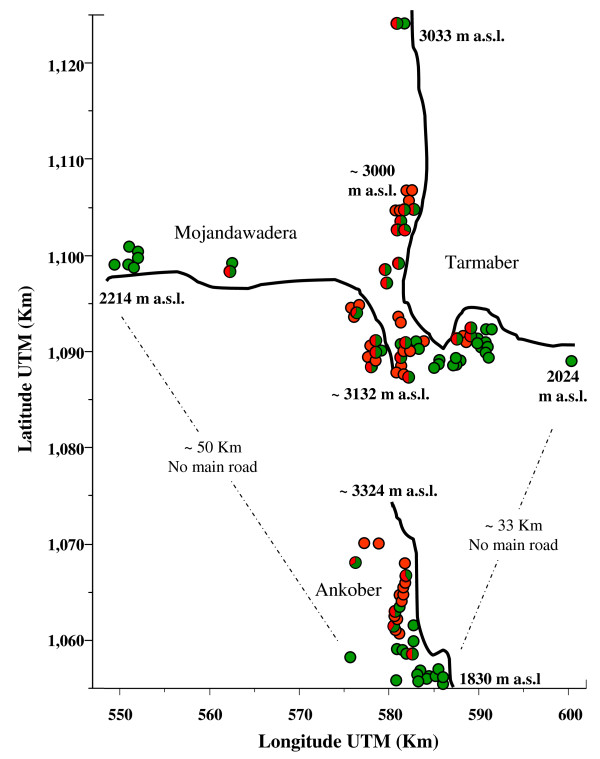
**Map of the collection sites**. Collection site coordinates and average *q *values for clusters S1 (green) and S2 (red) of each of the barley landraces. Landraces that showed an average *q *> 0.70 were considered completely assigned to one of the two clusters identified by STRUCTURE.

Figure 1 shows the collection site coordinates and the average *q *values for clusters S1 and S2 of each barley landrace.

**Figure 2:**
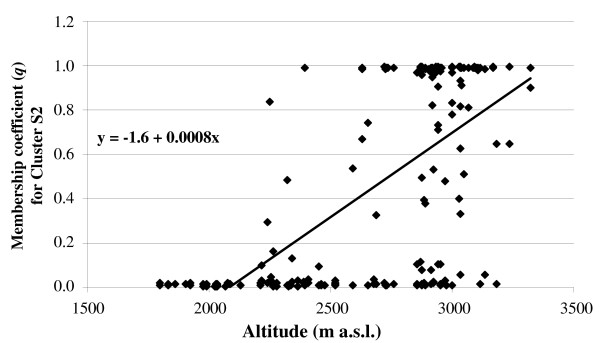
**Linear regression analysis for altitude and membership coefficient (*q*) for cluster S2 identified by STRUCTURE**.

**Figure 3:**
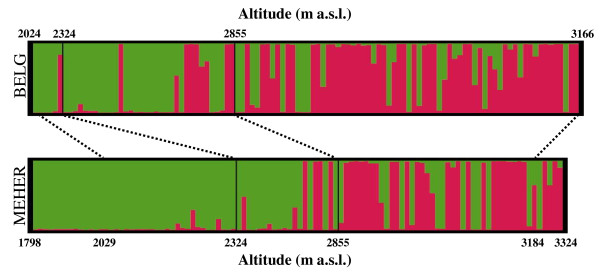
**Population structure across seasons and altitudes using STRUCTURE**. STRUCTURE assignment at *K *= 2 for the 108 individuals collected during the *Belg *growing season and the 104 individuals collected during the *Meher *season, ordered according to crescent altitude levels. Green, cluster S1; red, cluster S2.

**Figure 4:**
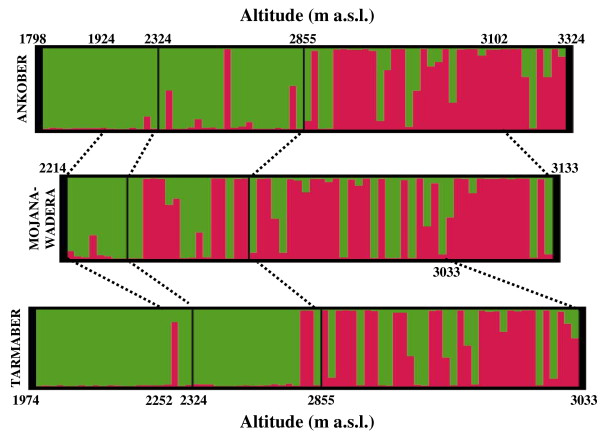
**Population structure across districts and altitudes using STRUCTURE**. STRUCTURE assignment at *K *= 2 for the individuals collected in the three districts (Ankober, Mojanawadera and Tarmaber), ordered according to crescent altitude levels. Green, cluster S1; red, cluster S2.

When the individuals were sorted according to altitude, the STRUCTURE analysis revealed a well-defined cline of variation; indeed, at low altitudes, cluster S1 was mainly seen, and this tended to be substituted by cluster S2 at high altitudes. The relationships between altitude and *q *for cluster S2 were assessed using a simple linear regression model. This regression was significant (R^*2 *^= 0.42, F = 152.5 and P < 0.0001; Figure 2), and it was consistent within the *Meher *(R^*2 *^= 0.45, F = 85.0 and P < 0.0001) and the *Belg *(R^*2 *^= 0.36, F = 59.4 and P < 0.0001) seasons (Figure 3), and within the three districts (P < 0.0001, R^2 ^= 0.54, 0.21, and 0.40, F = 83.6, 17.8, and 50.0; for Ankober, Mojanawadera and Tarmaber, respectively) (Figure 4).

**Figure 5:**
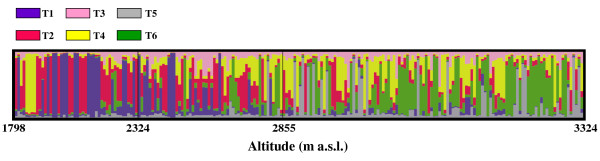
**Population structure along the altitude gradient using TESS**. TESS assignment at *K *= 6 for the 212 individuals, ordered according to crescent altitude levels. Blue-purple, T1; red, T2; pink, T3; yellow, T4; grey, T5; green, T6.

**Figure 6:**
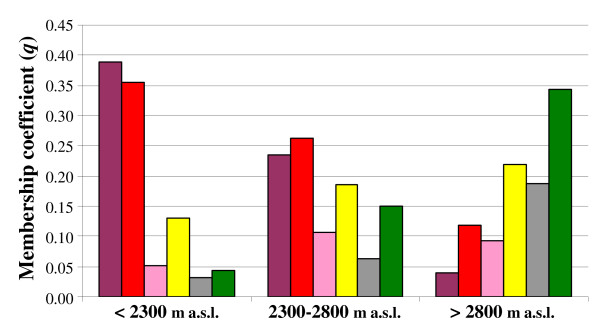
**Average membership coefficient (*q*) for the six TESS clusters for the three altitude classes**. See legend to Figure 5 for colour key.

The second approach used for the population structure analysis was the algorithm implemented in the TESS software that allows for prior spatial information when identifying clusters of individuals. This TESS software allows the testing of different values of the spatial dependent parameter (*Ψ*); this parameter weights the relative importance given to spatial connectivities. We tested three different *Ψ: *0.0 (no spatial correlation between individuals), and 0.6 and 1.0 (moderate and strong spatial correlation between individuals, respectively). For each *Ψ *value considered, we performed 100 runs for *K *from 2 to 10. Then the DIC values of the 10 runs with the smallest DIC were averaged. In plotting the average DIC values *versus K *(from 2 to 10), we obtained an inflection point at *K *= 6. This was the same for all of the different interaction parameter (*Ψ*) values considered. Thus the best model was obtained for *K *= 6 and the lowest DIC value corresponded to *Ψ *= 0.6, indicating a moderate spatial correlation between individuals. The average over 10 runs with the smallest DIC value *q *was computed. We defined the six TESS clusters as T1, T2, T3, T4, T5 and T6. One hundred and twenty-five individuals (59%) were assigned to one of the six clusters with a *q *higher than 0.60, with 104 (49%) higher than 0.70, and 70 (33%) higher than 0.80. Figure 5 shows the assignment results for all of the individuals when ordered according to altitude. Averaging the *q *for the six TESS clusters for the three altitude classes, we observed that the previous cline of variation along altitude identified by the STRUCTURE assignment was also maintained for TESS; indeed, at low altitudes, the T1 and T2 clusters were mainly present, and these tended to be substituted by the T5 and T6 clusters at high altitudes (Figure 6). The T3 cluster was mostly present at intermediate and high altitudes, while T4 was present in all of the altitude classes (Figure 6). However, the genotype assignment for T4 is more scattered at intermediate and high altitudes than at low altitudes (Figure 5). Indeed, at low altitudes, this genotype assignment for T4 is characteristic of two populations collected during the *Meher *season. It is important to note that the percentage of individuals that had a *q *to one of the six TESS clusters higher than 0.70 is highest (76%) at low altitudes (<2,300 m a.s.l.), and decreases at intermediate (35%) and high (45%) altitudes.

The correlation analysis using Spearman's rho (ρ) for non-parametric tests showed that cluster S1 identified by STRUCTURE was significantly correlated with TESS clusters T1 and T2, and cluster S2 with T5 and T6 (Additional file 3). The step-wise multiple regression analysis showed that mainly TESS clusters T1, T2, T3 and T4 provide a better prediction for cluster S1 (R^2 ^= 0.84), and T4, T5 and T6 are the major contributors for cluster S2 (R^2 ^= 0.84) (Additional file 4); TESS cluster T4 contributes to both of the S1 and S2 clusters identified by STRUCTURE. These results are consistent with the relationships between the altitude and the *q *for the TESS groups, which was tested by performing a simple regression analysis; indeed this relationship was highly significant (P < 0.0001) for the TESS clusters T1, T2, T5 and T6 (R^2 ^= 0.25, 0.14, 0.10, 0.22 respectively), significant (P = 0.05) for T3, but not significant for T4 (P = 0.08).

The ANOVA analysis revealed significant correlations between all of the loci considered and the altitude (Additional file 5).

Considering the morphological data, 82% of the character states considered and studied by Tanto Hadado *et al. *[16] were significantly correlated with altitude (P < 0.05), 79% of which had a probability lower than 0.01; moreover, at least one character state for each of the morphological traits considered was significantly correlated with altitude at P < 0.01 (Table 5).

**Table 5:** Relationships between frequencies of the character states for each population and altitude (Spearman's rho).

Character	**Character state**^**1**^	Spearman's ρ	**R**^**2**^	P
**Kernel row number**	Irregular	0.31	0.10	**0.001**
	Six rowed	-0.17	0.03	0.09

**Spike density**	Lax	0.02	0.00	0.81
	Intermediate	0.37	0.14	**0.0001**
	Dense	-0.48	0.23	**1.6e**^**-07**^

**Lemma awn barb**	Intermediate	0.35	0.12	**0.0002**
	Rough	-0.34	0.11	**0.0004**

**Glume colour**	White	-0.54	0.29	**3.3e**^**-09**^
	Brown	0.62	0.38	**2.0e**^**-12**^

**Lemma type**	No lemma teeth	0.24	0.06	**0.01**
	Lemma teeth	-0.24	0.06	**0.01**

**Length of rachilla hair**	Short	0.51	0.27	**1.6e**^**-08**^
	Long	-0.51	0.27	**1.6e**^**-08**^

**Kernel colour**	White	-0.53	0.28	**6.3e**^**-09**^
	Tan/red	0.39	0.16	**2.5e**^**-05**^
	Purple	0.15	0.02	0.13
	Black/grey	0.39	0.15	**4.2e**^**-05**^

Morphological data from Tanto Hadado *et al. *[16].

^1 ^Character states with frequencies lower than 0.05 and higher than 0.95 are not included in the analysis.

### Association between morphological and molecular characterisations

To investigate the correspondence between the morphological and molecular characterisations, a contingency analysis was performed. The genetic groups defined by TESS analysis were considered, and only the genotypes that were assigned to one of the groups with a *q *higher than 0.70 were included in the analysis (104 genotypes in all). No inference was carried out for TESS cluster T3 because no genotypes were assigned to this group according to this threshold. The analysis was conducted considering the seven morphological traits: kernel-row number, spike density, lemma awn barbs, glume colour, lemma type, length of rachilla hair, and lemma colour [16].

The results show that all of the traits except lemma awn barbs (P = 0.066), were significantly associated with the TESS genetic clusters. The association was higher for length of rachilla hairs, glume colour and spike density (P < 0.0001), followed by lemma colour (P < 0.001), row number (P = 0.003) and lemma type (P = 0.011). The proportion of variance of each morphological trait explained by genetic groups (R^2^) varied from 36.6% (length of rachilla hairs) to 13.0% (lemma type), with an average of 22.5%. The types and frequencies of each of these seven qualitative traits in each of the TESS clusters (with the exclusion of T3, as indicated above) are given in Additional file 6.

The relationships between the genetic groups according to morphological characterisation were assessed using a non-parametric correlation analysis (Spearman's rho) (Additional file 7). The clusters T1 and T6 (the former mainly present at low altitudes, and the latter at high altitudes) showed the highest morphological differentiation. T1 included mainly six-rowed (65.2%) and irregular (21.7%) types, and was the only group that included two-rowed types (4.4%), and it showed the highest frequency among all of the groups for the two-rowed deficient types (8.7%). Both the regular and deficient two-rowed types were absent in T6, which included mainly irregular types (74.2%). T1 was characterised by a high frequency of dense spike types (47.8%), while T6 was characterised by lax types (41.9%), and both of these groups showed high frequencies of intermediate spike density types (43.5% and 54.9%, respectively). T1 was monomorphic for the lemma awn barbs (rough) and glume colour (white) traits; in contrast, T6 also showed intermediate lemma awn barb types (12.9%), and brown (35.5%) and black (6.4%) glume colour types. All of the individuals of T6 had short rachilla hairs, while those of T1 had both short (52.2%) and long (47.8%) rachilla hair types. Finally, differences were seen also for the lemma colour trait: T1 was mostly represented by white types (82.6%), while T6 was mostly represented by black types (74.2%).

### Spatial structure and landscape analysis

Mantel tests between the genetic and geographical distance matrices were significant (*r = *0.12, P < 0.001). However, the correlation between molecular and geographical distance was about 2-fold lower than that between the molecular distances and differences in altitude (r = 0.28, P < 0.001), and also lower than that between geography and altitude (r = 0.14, P < 0.001).

**Figure 7:**
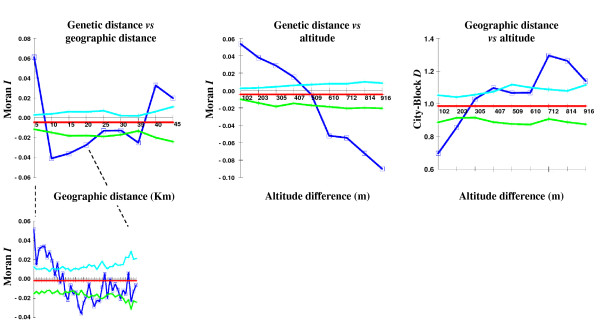
**Autocorrelation analysis**. Results of the autocorrelation analysis using all 212 genotypes and all loci. The 99% probability envelopes are indicated: light blue, upper limit; green, lower limit. Dark blue, observed data: for genetic distance *vs *geographical distance). Inset: the first 20 km from panel a) (40 classes, 500 m each).

The same pattern was seen for the spatial autocorrelation analysis, which showed that within a distance class of 5 km, positive and significant Moran's *I *values were detected. This indicates that the proximal individuals (included those of the same population/field) were more genetically similar (i.e. related) than expected from a random distribution (Figure 7a). In this class, the individuals at similar altitudes within each district were compared. The degrees of correlation, however, rapidly decreased, and there were already negative Moran's *I *values for the 10-km distance class. In this class, individuals from relatively different altitudes were mostly compared, mainly within each district. A similar situation was seen in the 20-km and 30-km distance classes. The degrees of correlation, however, tended to increase, and interestingly, positive and significant Moran's *I *values were found for the 40-km and 50-km distance classes: when individuals of different districts but at similar altitudes (either low or high) were compared, they tended to be genetically more similar than for a random distribution (Figure 7a). This is particularly evident considering the relationships between the genetic and altitude distances; indeed, the individuals that were collected at similar altitudes showed higher similarities than those collected at different altitudes (Figure 7b).

Finally, it is important to note that based on the distribution of the collection sites, when individuals from similar altitudes were compared, they also tended to be geographically more distant. In contrast, the individuals from different altitudes were geographically closer (Figure 7c).

Spatial autocorrelation between geographical and genetic distances was also performed separately for the three altitude classes (Additional file 8), which confirmed the previous trends seen, showing at low altitudes (<2,300 m a.s.l.) a clear geographical effect (isolation by distance relationship among plants), while at high altitudes (>2,800 m a.s.l.), this effect disappeared, as all of the similarity values were not significantly different from random values, and thus no geographical structure was evident. A non-significant, intermediate (but as for the >2,800 m a.s.l. class) trend was seen at moderate altitudes (2,300-2,800 m a.s.l).

### Sliding-window analysis

**Figure 8:**
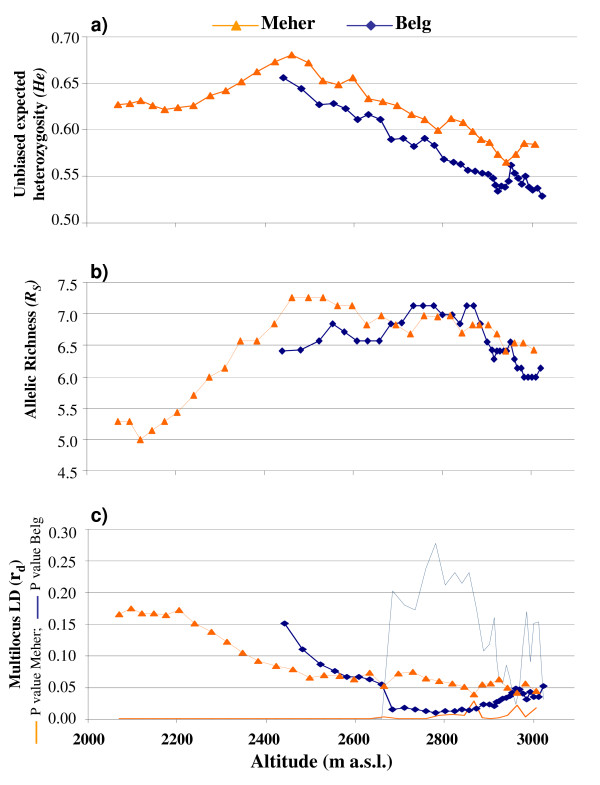
**'Sliding-window' analysis for the two seasons: *Belg *(blue) and *Meher *(orange)**. **a) **Genetic diversity (*He*); **b) **allelic richness (*R*_S_); **c) **multilocus linkage disequilibrium (LD) along the altitudinal cline, with P values also shown. The altitude of each window (20 populations, 40 individuals) was determined by averaging the altitude of the populations (farmers' fields); hence, the differences in the average altitudes between the windows are not constant.

Figure 8 shows the results of the 'sliding-windows' analysis. In Figure 8, each point represents: for the y axis, the *He*, *R*_*S *_and LD estimates of a group of 40 individuals from 20 landrace populations (window); and for the x axis, the mean altitude over all of the 20 populations. Thus, the diversity and LD estimates were computed along the altitudinal cline. This analysis was carried out separately for each of the two seasons. The *Belg *season was not represented at low altitudes, while the *Meher *season was characterised by the whole altitude range considered.

To test the significance of the trends seen, we grouped the individuals into different and non-overlapping altitude ranges: three for *Belg*: B1, B2 and B3 (average altitudes of 2,397, 2,859 and 3,022 m a.s.l., respectively), and four for *Meher*: M1, M2, M3, M4 (average altitudes of 1,962, 2,416, 2,909 and 3,138 m a.s.l., respectively). The significance was tested between adjacent altitude ranges, using the Wilcoxon signed-paired rank test and the Bonferroni correction. The choice of the altitude ranges was designed to have the average altitudes more uniform between the *Belg *(B1, B2, and B3) and the corresponding *Meher *(M2, M3, M4) classes (the M1 class did not have an equivalent in *Belg*, which was not represented at low altitudes in this analysis). The number of individuals per altitude range varied from 34 to 40 for *Belg*, and from 16 to 36 for *Meher*.

Considering the *Meher *season, the diversity (*He*) showed a significant increase according to altitude, to about 2,500 m a.s.l.. Indeed, M2 showed a higher level of diversity than M1 (Wilcoxon signed-paired rank test, after Bonferroni correction: P = 0.018); although no other comparisons were significant in the *Meher *season, a reduction at high altitudes was seen. A significant reduction of *He *at high altitudes was seen for the *Belg *season (B2-B3: P = 0.046) (Figure 8a).

The allelic richness, *R*_*S*_, showed a marked increase from low to intermediate altitudes in the *Meher *season (Wilcoxon signed-paired rank test, after Bonferroni correction: M1 < M2: P = 0.017), and reached a plateau at intermediate and high altitudes (M2 *vs *M3; M3 *vs *M4) (Figure 8b). For the *Belg *season, the trend of allelic richness resulted in a slight increase at intermediate altitudes (B1 < B2: P = 0.046) and a decrease at high altitudes (B2 > B3, P = 0.018).

The multilocus LD among our unlinked loci (Figure 8c) decreased with altitude for the season, and interestingly, only that for the *Belg *season reached non-significant levels in the interval between 2,685 and 3,010 m a.s.l. (Figure 8c).

## Discussion

Several studies have analysed the diversity of Ethiopian barley landraces. However, along with our previous study on the same barley landrace collection that was based on morphological data [16], for the first time the present study has considered an *ad-hoc *collection of barley landraces from Ethiopia that was obtained on a single-plant basis, and that also included the two main growing seasons.

### Level of polymorphism in Ethiopian barley landraces from North Shewa

In this study, we analysed a collection of barley landraces from an area of about 2,000 km^2 ^in the central highlands of Ethiopia, in North Shewa. All of the seven microsatellites used for the analyses are in common with the study of Russel *et al. *[44] which included barley landraces from several different agro-ecological areas of Syria and Jordan, and six are also shared with the studies of both Pandey *et al. *[36], which analysed barley landraces from the highlands of Nepal, and Macaulay *et al. *[37], which analysed a sample of modern barley varieties. Interestingly, as the different barley samples were analysed with the same microsatellites, this has allowed us to make comparisons between the results of the present study and those obtained in these other studies. We have shown here a relevant level of genetic diversity in the Ethiopian barley landrace collection, which was higher than both the Nepalese barley landraces and the sample of modern varieties. The substantial amount of genetic variation detected in the present study was also in agreement with the high level of morphological diversity seen in the same sample of landrace populations by Tanto Hadado *et al. *[16].

The Ethiopian landraces from North Shewa showed a lower genetic variation than those of the Syrian and Jordanian landraces. However, it is important to consider that Russel *et al. *[44] worked at a country level (i.e. on a larger geographical scale), with their study of barley landraces collected from several different agro-ecological areas of Syria and Jordan. For this reason, the level of diversity found in the much smaller area in our survey in the present study is undoubtedly remarkable.

### Population structure

Overall, the population structure appears to be very low between seasons and geographical districts, while it is highly associated with altitude differences. Indeed, we show significant, but very low, divergence (*F*_ST _= 0.02) with no private alleles (*p *< 0.05) between the two different planting seasons. Moreover, the two seasons show very similar levels of diversity. These data are very consistent to those for the morphological traits on the same populations [16], and they confirm that seed flow occurs between seasons, as was also suggested by 5% of the farmers' fields during the collection, where a sequential cultivation of barley was seen in the two seasons. In other words, barley is often planted in one season using the seeds obtained from the harvest in the previous season. A slightly higher divergence was seen between districts, with Ankober having a higher diversity compared to the other two districts.

In contrast, the divergence estimators show strong effects due to altitude. In the present study, we used two different Bayesian approaches (the STRUCTURE and TESS programmes) to make inferences on the population structure of our sample. STRUCTURE identified two main clusters: rather than a subdivision among seasons or districts, these two clusters highlighted a clear altitudinal cline, and this tendency was maintained both within seasons and districts. Even though the analysis conducted with TESS identified more genetic groups (six), these were in line with the STRUCTURE results. Indeed, five TESS clusters were highly associated to the two clusters identified by STRUCTURE, with only one exception: TESS cluster T4. This indicates that the data obtained with the two different approaches are not completely redundant, with a more detailed and refined picture being provided by TESS. However, independent of a clear or a less clear definition of the "real" numbers of the populations that characterised our sample of genotypes, it is important to stress that both of these tools were very useful and that they were in agreement in the defining of the major aspects of the genetic structure of these barley landrace populations. For instance, as for the STRUCTURE clusters, the TESS groups reflect the altitudinal cline of variation, with five of them significantly correlated with altitude. The same effect of altitude was also seen for all of the SSR loci used in the present study. Moreover, most (84%) of the character-state frequencies obtained from Tanto Hadado *et al. *[16] (at least one for each trait) were found to be significantly associated with altitude. Thus, in line with other published works cited in the Introduction, the present study has identified a strong association between altitude and genetic composition of barley landraces from Ethiopia, with a clear altitudinal cline shown.

Two possible evolutionary scenarios can be considered to explain the altitudinal cline of variation that we have detected here: a clinal variation might have originated by various patterns of migration, or by different selections along the geographical or environmental gradients [45]. The results in the present study for the spatial autocorrelation between altitude and genetic distances reflects a continuous and constant reduction in the Moran *I *index, from positive to negative values; such a trend is characteristic of a typical strong selection gradient [45]. However, when there is a large difference between the immigrant and the resident gene frequencies, this trend can also be generated by a specific case of unidirectional migration [45]. The hypothesis of unidirectional migration in our case would be in a vertical direction, from low to high altitudes, although even if this is theoretically possible, under our conditions it is not realistic. This appears evident from the geographical structure of the collection area, with the three districts. Moreover, when we analysed the correlogram related to the comparison between geographical and genetic distances, for the first distance class (between genotypes at a distance lower than 5 km), there were positive Moran *I *values that became significantly negative just between the genotypes with a distance of 5 km to 10 km, where the differences in altitude are already substantial (on average, 125-250 m). Furthermore, the Moran *I *values became positive only at high geographical distances, when comparisons were mainly made between genotypes at similar altitudes, but in different districts. These data confirm the hypothesis that selection for adaptation at different altitudes is the main factor that determines the clinal variation seen. This divergence between different districts but within the same altitude class is consistent with the selection hypothesis, and it is suggestive of different selection intensities at low and high altitudes. Indeed, when we compared individuals at low altitude across the three districts, there was a significant and relatively high *F*_ST _(0.10, P < 0.01), while there were very low and non-significant *F*_ST _values between individuals at intermediate (0.04) and high (0.01) altitudes from different districts. These data are confirmed by a separate spatial autocorrelation at different altitude classes, which shows a correlation between geographical distances and genetic distances only at low altitudes. This suggests that the homogeneous selection for adaptation to high altitudes is the major factor that explains these data, along with isolation by distance (IBD) at low altitudes. Alternatively, we should consider the hypothesis of different migration patterns at low *vs *high altitudes: a higher level of seed flow between districts at high altitudes than at low altitudes.

In support of the role of adaptive selection in shaping the allele frequencies of our collection, we found that the two morphological traits analysed that relate to colour (kernel and glume colour) are significantly associated with altitude. With their colour linked to the presence of anthocyanins, the coloured types in particular were positively related to altitude, while the white types showed a negative relationship with altitude. These anthocyanins are a subgroup of the flavonoids, for which an important role is well documented in responses to both biotic and abiotic stresses (see [46], for review). Thus, the higher presence of coloured types at high as opposed to low altitudes might be an adaptive response to the increased UV-B radiation characteristics at high altitudes.

However, even though there is a large amount of coherent evidence that suggests that selection is a crucial factor in shaping the diversity of Ethiopian barley landrace populations, particularly at high elevations, selection alone is not enough to explain the diversity patterns seen. Indeed, the diversity significantly increases from low to moderate altitudes (2,500 m a.s.l.), both in its richness and its expected heterozygosity. This occurred only for the *Meher *season, because barley is grown in the *Belg *season mainly at higher altitudes. This pattern can be explained by the increasing number of populations (fields) and by the higher population sizes (larger plots), as shown by Tanto Hadado *et al. *[16]. At low altitudes, the environmental conditions are more favourable on average, and many crops can be grown easily; thus the farmers tend to grow many different crops (e.g. tef, maize and sorghum), and in general, barley tends to be cultivated in smaller plots, as compared to at higher elevations. Thus, the increased diversity might be related to the greater population size of the barley landraces, which is not only related to the barley plot size increase, but also to the two seasons of barley growth per year at intermediate and high altitudes; thus, the effective number of generations per year is higher than one [16]. The maximum diversity is seen at moderate altitude (2,460 m a.s.l.), where due to the spring rains, the barley is grown in both seasons. When the altitude further increases, for both seasons, the diversity measured as expected heterozygosity decreases significantly with altitude, while the richness remains almost constant, even if at very high altitudes a small but significant reduction is seen. This trend is in agreement with the hypothesis of an increasing selection intensity for adaptation at high altitudes. Selection is expected to increase the frequency of favourable alleles, which would reduce the expected heterozygosity, while the richness is less affected by selection, compared to the expected heterozygosity; this would be favoured by the greater population size. However, at very high altitudes, a reduction in the number of alleles is seen. Thus, we explain the diversity pattern seen by the combined effects of selection and population size. While the plot (field) sizes constantly increase with altitude in a linear fashion, the number of fields increases up to 2,800-3,000 m. a.s.l., followed by a reduction at higher altitudes [16]. Thus, the overall population size increases exponentially according to altitudes up to 2,800-2,900 m a.s.l., with a reduction at higher altitudes.

Our interpretation is further supported by the reduction in the multilocus LD that was seen from low to high altitudes, which was even non-significant in the *Belg *season, while at the extreme altitudes, the multilocus LD increased slightly. The reduction in the LD might also be explained by both the greater population size, which would increase the effective recombinations, and the selection, which will favour recombination that will produce novel multilocus genotypes.

The level of observed heterozygosity was very low (0.003), in agreement with the strict selfing nature of barley where the level of outcrossing is lower than 1-2% [47-50]. Only three individuals, all located at high altitude, showed one or two heterozygous loci. Clearly, our study does not have the power to discriminate between different altitudes for the level of heterozygosity. However, a higher level of heterozygosity at high altitude might partially explain the pattern of LD in our study, because of a higher effective recombination rate at high altitude. To test this hypothesis a further study should be conducted using a larger sampling.

## Conclusions

Landraces are a key component of agro-biodiversity, and they represent a crucial reservoir of genetic diversity for plant breeding. Moreover, *in-situ *conservation of landraces can provide a number of advantages, including the potential for adaptation to environmental changes because of an ongoing evolutionary process. Nevertheless, few studies have described the population structures of landraces or have analysed the roles of different evolutionary forces in the shaping of their genetic diversity. Here, we show that barley landraces from Ethiopia have highly variable local populations (farmer's fields) with large within-population diversity. These landraces also appear to be locally adapted, and the major driving force that has shaped their population structure is selection for adaptation along an altitudinal gradient. Moreover, our data suggest that the two-season system (which characterises barley cultivation in Ethiopia) and its effects on landrace population size have important roles in counterbalancing the homogenising effects of selection. Our study highlights the potential of barley landraces from Ethiopia as a source of useful alleles. They are also an ideal system to study the processes of adaptation and for identification of genes and genomic regions that have adaptive roles in a crop species, which can be achieved through association mapping and scanning for signatures of selection for molecular-diversity structure.

## Methods

### Plant materials

The plant materials were derived from a barley landrace collection that was carried out in 2005, in the North Shewa zone in the central highlands of Ethiopia [16]. This collection [16] was obtained by visiting the same three districts (Ankober, Mojanawadera and Tarmaber) in both the long (*Meher*) and the short (*Belg*) growing seasons. Within each farm visited, and according to the information from the farmers, all of the different landraces of barley that were grown and kept separated by the farmers during the seed selection process were collected separately from different fields and considered as different landrace populations (including those sampled in different seasons from the same farmer). In most cases (80 farmers), only one landrace was grown, while 10 of the farmers grew two landraces, and only two grew three landraces. For this reason, per farmer, more than one landrace population was sampled (on average, 1.15). Overall, the collection includes 106 barley landrace populations (fields), and Additional file 9 shows the collection site coordinates of these barley landrace populations. Within each field, 100 spikes (one spike per plant) were randomly sampled all along a diagonal of the field, with the plants sampled from 5-10 m apart. The geographical position of each field (as latitude, longitude and altitude) was determined using the Global Positioning System (GPS). Before threshing, 30 spikes per population were randomly sampled from the 100 spikes collected from each field. The morphological evaluation of these materials was based on eight morphological traits of the mature spikes (kernel-row number, spike density, lemma awn barbs, glume colour, lemma type, length of rachilla hair, kernel cover, and lemma colour), as reported by the Bioversity International Barley descriptors [51]; the details of the sampling strategy and morphological evaluation are given in [16].

From this collection, we randomly sampled two individual spikes for each population (farmer's field) collected from different individual plants. From each spike, a single seed was grown to the three-leaf stage and used for DNA extraction. Overall we analysed 212 genotypes from 106 barley landrace populations.

### Genotypic data

Seven SSR markers (HVM20, Bmac0134, Bmag0013, HVM67, Bmac0113, Bmac0040, Bmac0156), as one marker per chromosome (see Additional file 10), were selected from Ramsay *et al. *[52] and used for the genetic characterisation of the 212 genotypes considered. The DNA was obtained from young leaves of single plants, using the miniprep extraction method of Doyle and Doyle [53]. The amplification conditions are reported in Additional file 10. The genotyping of the seven SSR markers was carried out with the ABI Prism 3100-Avant Genetic Analyser automatic sequencer, with GENESCAN 7.0 analysis software (PE Applied Biosystems, Foster City, CA, USA).

### Statistical analysis

#### Level of polymorphism

The number of alleles (*n*_*a*_), the average number of observed alleles per locus (*n*_*o*_), the effective number of alleles per locus (*n*_*e*_, [54]), the Levene [55] observed heterozygosity (*H*_*o*_) and the Nei's [35] unbiased genetic diversity (*He*) estimates based on allele frequencies, were calculated for each SSR locus. These were analysed as averages for each of the seasons (*Belg *and *Meher*), the districts (Ankober, Mojanawadera, Tarmaber), and the three altitude classes (<2,300, 2,300-2,800, >2,800 m a.s.l.), and for the whole sample, using the POPGENE software, version 1.31 [56]. As the number of alleles observed was highly dependent on the sample size, we also computed the allelic richness (*R*_*S*_, [57]), using the FSTAT software [58], a methodology that estimates the number of alleles independent of the sample size. The number of private alleles and their average frequencies for each of the above-mentioned groups were determined by inspection of the allele distributions. This computation was carried out also considering a minimum threshold frequency of 5%, to reduce the effects of sampling error [59]. The differences between seasons, districts, and altitude classes for the genetic diversity estimates (*n*_*a*_, *n*_*e*_, *He *and *R*_*S*_) were tested using the Wilcoxon signed-ranks non-parametric test for two groups, arranged for paired observations (i.e. one pair of estimates for each locus) [60].

The partitioning of the genetic diversity was obtained using an analysis of molecular variance framework (AMOVA, [61]). This AMOVA analysis was performed using the Arlequin software, version 3.1 [62]. The divergence between seasons, districts, and altitude classes for each of the SSR loci was analysed, and the averages were quantified with *F*_ST _[63] and *R*_ST _(R-statistics, [64]) estimators. *F*_ST _and *R*_ST _differ in sensitivity when estimated on SSRs [65]; indeed *F*_ST _can underestimate the magnitude of differentiation when populations are highly structured or are in a situation where the SSRs show high mutation rates; in contrast, *R*_ST _is independent of the mutation rate, even if it has a high associated variance. We also considered the three altitude classes separately within each district, and we performed AMOVA analysis to compute the average unweighted *F*_ST _estimates between different districts within the same altitude classes (<2,300, 2,300-2,800, >2,800 m a.s.l.). Similarly, the pairwise *F*_ST _estimates between districts within the same altitude classes were computed.

All of the seven SSRs used were the same as those for the study of Russel *et al. *[44], who worked on barley landraces from several different agro-ecological areas of Syria and Jordan. Similarly, six (excluding HVM20) were common to the studies of Pandey *et al. *[36], who worked on Nepalese barley landraces, and Macaulay *et al. *[37], who worked on a set of modern barley varieties. This has allowed us to make comparisons among and between the diversity levels detected previously in Ethiopian, Syrian, Jordanian and Nepalese landraces, and with modern varieties of *Hordeum vulgare*.

#### Population structure

To further investigate the population structure of our sample, a Bayesian-model-based approach was used, as proposed by Pritchard *et al. *[40] and implemented in the software STRUCTURE, version 2.1 [41], to assign the genotypes into genetically structured groups. The software was run for presumed populations (*K*) from 1 to 10, following the admixture ancestry model. The run length was 100,000 MCMC repetitions and 100,000 burn-in periods, with 100 independent replicates for each *K*, to achieve consistent results. An *ad-hoc *statistic (Δ*K*, [43]) was used to estimate the "true" *K *number. The percentages of membership (*q*) of the individuals in each of the inferred *K *clusters were computed by an additional run using 1,000,000 MCMC repetitions and 1,000,000 burn-in periods.

An additional cluster analysis was performed with TESS, version 2.0 [42], a programme that introduces spatial correlation between individuals, in contrast to STRUCTURE, which assumes that all of the individuals are equally unrelated. The incorporation of a spatial component into the clustering model has the potential to determine if the clines provide a sensible description of the underlying pattern of variation [66-68]. TESS implements a Bayesian clustering algorithm that uses a hidden Markov random field (HMRF) model to compute the proportion of individual genomes originating in *K *populations. The HMRF represents spatial connectivities as links in a network of individuals. Furthermore, it incorporates decay of the membership coefficient (*q*) correlation with distance, a property similar to isolation-by-distance. The network was automatically generated by the TESS programme using a Dirichlet tessellation obtained from the spatial coordinates of the samples. Considering the spatial distribution of our samples, we modified the network by removing several links, to account for potential geographical barriers. Runs were based on a burn-in period of 20,000 cycles followed by 30,000 iterations. One hundred replicates were performed for *K *values from 2 to 10, and for all of the runs the admixture model was used. In the analysis, we considered three values of the spatial dependence parameter (*Ψ*), 0.0, 0.6 and 1.0. This parameter weights the relative importance given to the spatial connectivities (*Ψ *= 0 recovers the model underlying STRUCTURE, while *Ψ *= 0.6 and 1.0 indicate moderate and strong values, respectively). For each run, the programme computed the Deviance Information Criterion (DIC), which is a model-complexity-penalised measure of how well the model fits the data. The lower DIC values represent the better fits for the data. Thus, we selected the 10 runs for each *K *that corresponded to the 10 lowest values of the DIC, and we averaged these values to determine the 'true' *K *number.

The TESS algorithm incorporates an additional regularisation feature that generally leads to a less ambiguous determination of *K *[42,68]. Moreover, it can achieve an accuracy similar to that obtained with non-spatial methods, while using a smaller number of genetic markers [68]. Thus, we averaged the estimated *q *over the 10 runs with the smallest values of the DIC for the *K *value identified. We used the software CLUMPP, version 1.1 [69], which implements the *Greedy *algorithm, to allow for label switching and to decide which of the clusters of each run corresponded to a specific label.

The results of both of the STRUCTURE and TESS Bayesian clustering programmes were visualised using DISTRUCT, version 1.1 [70]. The output represents each individual as a vertical line, partitioned into *K *coloured segments, which represent the individually estimated membership fractions in the *K *clusters.

A simple linear regression model was used to test the relationships between the altitude and the *q *for the STRUCTURE and TESS clusters. The *q *values for STRUCTURE and TESS were used to investigate the relationships between the clusters identified by these two different programmes. The analysis was performed using Spearman's rho (ρ) for non-parametric correlation. Moreover, a step-wise multiple regression model was also performed, with the STRUCTURE clusters as dependent variables and the TESS clusters as independent variables. These analyses were carried out using the JMP 7 software (SAS Institute, Cary, USA).

#### Association between the genetic and morphological characterisations

To investigate the associations between the genetic and morphological characterisations, a contingency analysis was performed with the likelihood ratio chi-squared test, using the JMP 7 software. The proportion of the total uncertainty attributed to the model fit (R^2^) was also calculated. For this analysis, we considered the TESS genetic groups and only the genotypes assigned to one of the groups with a *q *higher than 0.70. No naked (hulless) barley was present among the 212 individuals analysed, thus the analysis was conducted considering seven morphological traits: kernel-row number, spike density, lemma awn barbs, glume colour, lemma type, length of rachilla hair, and lemma colour [16].

#### Spatial structure, clinal variation and landscape analysis

Spatial autocorrelation between spatial (geographical and altitude) and genetic distances were computed separately using Spatial Genetic Software (SGS), version 1.0 d [71]. These calculations were carried out using Moran's *I *[72,73] for spatial distance classes (in metres), the dimension of which was 5,000 m for geographical distances (9 classes), and 102 m for altitude differences (9 classes). The sizes and numbers of the classes were fixed, to retain biological meaning and to guarantee at least 1,000 pairs of data points in each class. The significances of the observed average Moran's *I *values were assessed by comparing them with the corresponding values derived by randomly permuting the multilocus genotypes over the spatial coordinates of the samplings (500 times). The 99% confidence envelopes were estimated.

We separately obtain the Moran's *I *correlograms over all of the seven SSRs and for each locus. Moreover, we also designed the distogram between geographical distance and altitude difference by considering the former as a quantitative trait and the latter as a linear geographical distance, and using the city-block distance.

The AIS software [74] was used to calculate the associations between genetic distances among individuals and geographical and altitude distances, using a Mantel test [75]. The genetic distances between individuals were calculated following Equation 3 of Miller [74], which is similar to Nei's [76] measure of genetic distance, except that allelic similarities are measured between individuals rather than populations.

To test for correlation between the SSR alleles and altitude, an analysis of variance (ANOVA) was performed with the Wilcoxon non-parametric test. Moreover, using the morphological data of the Tanto Hadado *et al. *[16] study (thus 106 populations, 30 individuals per population), we computed the frequencies of the characters for each population (character states with a frequency lower than 0.05 and higher than 0.95 were not included in the analysis), and we tested their relationships with altitude using the Spearman's rho (ρ) for non-parametric correlations. These analyses were carried out using the JMP 7 software.

#### Sliding-window analysis

To obtain a representation of the total genetic structure of the Ethiopian barley landraces from North Shewa that is as accurate as possible, we performed a sliding windows analysis. This approach is commonly used in ecology [77,78], but, to the best of our knowledge, has not used in molecular analyses. This allowed us to represent the variations of the genetic diversity (*He*), the allelic richness (*R*_S_), and the linkage disequilibrium (LD) along the altitudinal cline.

We reconstructed populations of 20 landraces (fields), which corresponded to 40 individuals (windows); each window differed by only one landrace population (two individuals), that represented the step size, moving forward from the lowest to the highest 20 landrace populations along the altitudinal gradient. Thus, considering the step size of one population, the windows partially overlapped, sharing 38 individuals (19 landrace populations). This analysis was carried out separately for the two seasons. The altitudes for each window were obtained by averaging the altitudes of the populations (farmers' fields) included in the window. Thus the differences in altitudes between the windows varied along the altitudinal cline.

To examine the LD (non-random association between alleles at different loci) between our unlinked loci, a multilocus summary statistic of association between loci, r_d_, was calculated [79]. The significance of its deviation from zero was obtained using the MultiLocus software, version 1.2 [80], which performs shuffling of genotypes with each locus and the overall loci. The expectation of this statistic is that it is independent of the number of polymorphic loci within the population examined, allowing comparisons between different samples.

To test the significance of the trends observed for *He *and *R*_*S*_, we grouped the individuals following three altitudinal ranges for *Belg*, and four for *Meher*. The differences were tested between adjacent altitudinal ranges within each season using the Wilcoxon signed-ranks non-parametric test [60].

## Authors' contributions

RP conceived, designed and coordinated the study; TTH and DR carried out the barley landrace collection in Ethiopia; EB performed the DNA extraction and molecular analysis; TTH DR EB and RP analysed the data and contributed to the drafting and writing of the manuscript. All of the authors have read and approved this version of the manuscript.

## Authors' Information

This study is a component of the PhD thesis of Tesema Tanto Hadado, which focused on an analysis of phenotypic and molecular diversity in barley landraces from the central highlands of Ethiopia. All of the contributing authors are broadly interested in molecular evolution and ecological genetics, with particular interests in the conservation and use of biodiversity and crop genetic resources.

## Supplementary Material

Additional file 1 **Summary statistics computed for each locus considering the two seasons, three districts, and three altitude classes, and for the whole sample**. Click here for file

Additional file 2 **Divergence (*F*_ST _and *R*_ST_) estimates for each of the SSR loci analysed, computed considering the seasons, districts, and altitude classes**. Click here for file

Additional file 3 **Non-parametric correlation (Spearman's rho) between TESS and STRUCTURE clusters**. Click here for file

Additional file 4 **Step-wise multiple regression analysis for STRUCTURE and TESS**. The model was performed considering the STRUCTURE clusters as dependent variables and the TESS clusters as independent variables, from the data illustrated in Figures 4 and 5. Click here for file

Additional file 5 **Relationships between the molecular data and altitude**. Click here for file

Additional file 6 **Types and frequencies of each of the seven qualitative traits, computed considering the genotypes that were assigned to one of the TESS clusters with a coefficient of membership (*q*) higher than 0.70 (from the data illustrated in Figure **5). Click here for file

Additional file 7 **Non-parametric correlation (Spearman's rho) between the TESS clusters for their morphological traits (from the data illustrated in Figure **5**)**. Click here for file

Additional file 8 **Spatial autocorrelation analysis between the geographical and genetic distances, performed separately for the three altitude classes. See legend to Figure **7** for colour key**. Click here for file

Additional file 9 **Collection site coordinates of the barley landraces analysed**. Click here for file

Additional file 10 **List of SSRs used in the present study**. Click here for file
