# Supplementary material for: Adaptation and diversity along an altitudinal gradient in Ethiopian barley (Hordeum vulgare L.) landraces revealed by molecular analysis
Source: BMC Plant Biol. 2010 Jun 21;10:121. doi: 10.1186/1471-2229-10-121 (PMC3095281; doi:10.1186/1471-2229-10-121)
Supplement: Additional file 1 — Summary statistics computed for each locus considering the two seasons, three districts, and three altitude classes, and for the whole sample. [file 1471-2229-10-121-S1.DOC]

**Additional file 1.** Summary statistics computed for each locus considering the two seasons, three districts, and three altitude classes, and for the whole sample.

|  |  | **Season** | | **District** | | | **Altitude class (m a.s.l.)** | | | **ALL** |
| --- | --- | --- | --- | --- | --- | --- | --- | --- | --- | --- |
|  |  | *Belg* | *Meher* | Ankober | Mojanawadera | Tarmaber | <2300 | 2300-2800 | >2800 |  |
| **HVM20** | *S* | 108 | 104 | 72 | 64 | 76 | 46 | 54 | 112 | 212 |
|  | *n_a_* | 3 | 4 | 3 | 4 | 3 | 3 | 3 | 3 | 4 |
|  | *n_e_* | 1.37 | 1.56 | 1.43 | 1.25 | 1.65 | 2.13 | 1.50 | 1.20 | 1.46 |
|  | *H_e_* | 0.27 | 0.36 | 0.30 | 0.21 | 0.40 | 0.54 | 0.34 | 0.17 | 0.32 |
|  | *R_S_* | 3.00 | 4.00 | 2.98 | 4.00 | 2.96 | 3.00 | 2.97 | 2.87 | 2.75 |
| **Bmac0134** | *S* | 104 | 100 | 67 | 63 | 74 | 46 | 54 | 104 | 204 |
|  | *n_a_* | 11 | 8 | 9 | 8 | 8 | 6 | 7 | 10 | 11 |
|  | *n_e_* | 5.28 | 4.17 | 5.28 | 4.43 | 3.67 | 2.07 | 4.10 | 3.90 | 4.98 |
|  | *H_e_* | 0.82 | 0.77 | 0.82 | 0.79 | 0.74 | 0.53 | 0.77 | 0.75 | 0.80 |
|  | *R_S_* | 11.00 | 8.00 | 8.98 | 8.00 | 8.00 | 6.00 | 6.97 | 9.22 | 7.55 |
| **Bmag0013** | *S* | 103 | 102 | 68 | 61 | 76 | 46 | 53 | 106 | 205 |
|  | *n_a_* | 6 | 6 | 6 | 6 | 6 | 6 | 6 | 6 | 6 |
|  | *n_e_* | 5.41 | 3.93 | 5.60 | 4.55 | 4.42 | 3.52 | 5.35 | 4.69 | 5.14 |
|  | *H_e_* | 0.82 | 0.75 | 0.83 | 0.79 | 0.78 | 0.73 | 0.83 | 0.79 | 0.81 |
|  | *R_S_* | 6.00 | 6.00 | 6.00 | 6.00 | 6.00 | 6.00 | 6.00 | 5.99 | 5.93 |
| **HVM67** | *S* | 108 | 104 | 72 | 64 | 76 | 46 | 54 | 112 | 212 |
|  | *n_a_* | 4 | 3 | 4 | 3 | 3 | 2 | 4 | 3 | 4 |
|  | *n_e_* | 1.50 | 1.89 | 1.67 | 1.52 | 1.85 | 1.97 | 1.83 | 1.24 | 1.69 |
|  | *H_e_* | 0.33 | 0.48 | 0.41 | 0.35 | 0.47 | 0.50 | 0.46 | 0.19 | 0.41 |
|  | *R_S_* | 4.00 | 3.00 | 3.98 | 3.00 | 3.00 | 2.00 | 3.93 | 2.63 | 3.00 |
| **Bmac0113** | *S* | 107 | 104 | 72 | 64 | 75 | 46 | 54 | 111 | 211 |
|  | *n_a_* | 6 | 5 | 6 | 5 | 3 | 4 | 5 | 6 | 7 |
|  | *n_e_* | 1.64 | 2.16 | 1.94 | 2.08 | 1.62 | 2.78 | 1.80 | 1.61 | 1.88 |
|  | *H_e_* | 0.39 | 0.54 | 0.49 | 0.53 | 0.39 | 0.65 | 0.45 | 0.38 | 0.47 |
|  | *R_S_* | 5.99 | 5.00 | 5.96 | 5.00 | 3.00 | 4.00 | 4.97 | 5.14 | 4.17 |
| **Bmac0040** | *S* | 105 | 104 | 72 | 63 | 76 | 46 | 54 | 111 | 211 |
|  | *n_a_* | 11 | 10 | 10 | 10 | 8 | 7 | 9 | 9 | 11 |
|  | *n_e_* | 5.51 | 6.89 | 6.22 | 5.61 | 6.43 | 4.22 | 5.21 | 4.70 | 6.78 |
|  | *H_e_* | 0.83 | 0.86 | 0.85 | 0.84 | 0.86 | 0.78 | 0.82 | 0.79 | 0.86 |
|  | *R_S_* | 10.99 | 10.00 | 9.98 | 10.00 | 8.00 | 7.00 | 8.93 | 8.37 | 8.59 |
| **Bmac0156** | *S* | 106 | 101 | 70 | 63 | 73 | 44 | 52 | 110 | 206 |
|  | *n_a_* | 19 | 22 | 20 | 17 | 19 | 11 | 16 | 20 | 23 |
|  | *n_e_* | 12.26 | 11.20 | 11.91 | 13.27 | 7.39 | 5.09 | 11.36 | 12.34 | 12.80 |
|  | *H_e_* | 0.93 | 0.92 | 0.93 | 0.94 | 0.88 | 0.82 | 0.93 | 0.93 | 0.93 |
|  | *R_S_* | 18.99 | 21.99 | 19.78 | 17.00 | 18.82 | 11.00 | 15.91 | 17.60 | 14.58 |

*S*, sample size; *n_a_*, number of observed alleles; *n_o_*, average number of observed alleles per locus; *n_e_*, effective number of alleles per *locus*; *He*, unbiased expected heterozygosity; *R_S_*, allelic richness.
