# Supplementary material for: Adaptation and diversity along an altitudinal gradient in Ethiopian barley (Hordeum vulgare L.) landraces revealed by molecular analysis
Source: BMC Plant Biol. 2010 Jun 21;10:121. doi: 10.1186/1471-2229-10-121 (PMC3095281; doi:10.1186/1471-2229-10-121)
Supplement: Additional file 2 — Divergence (FST and RST) estimates for each of the SSR loci analysed, computed considering the seasons, districts, and altitude classes. [file 1471-2229-10-121-S2.DOC]

**Additional file 2** Divergence (*F*_ST_ and *R*_ST_) estimates for each of the SSR loci analysed, computed considering the seasons, districts, and altitude classes.

| **Locus** | **Season** | | **District** | | **Altitude class** | |
| --- | --- | --- | --- | --- | --- | --- |
|  | ***F*_ST_** | ***R*_ST_** | ***F*_ST_** | ***R*_ST_** | ***F*_ST_** | ***R*_ST_** |
| **HVM20** (ch.1H) | 0.00 | 0.01 | 0.03* | 0.04* | 0.08*** | 0.08** |
| **Bmac0134** (ch.2H) | 0.02* | 0.07** | 0.04** | 0.11*** | 0.18*** | 0.39*** |
| **Bmag0013** (ch.3H) | 0.05*** | 0.00 | 0.01 | 0.02 | 0.03** | 0.02 |
| **HVM67** (ch.4H) | 0.03* | 0.00 | 0.01 | 0.00 | 0.28*** | 0.00 |
| **Bmac0113** (ch.5H) | 0.01 | 0.00 | 0.01 | 0.01 | 0.03* | 0.01 |
| **Bmac0040** (ch.6H) | 0.03** | 0.02 | 0.01 | 0.01 | 0.09*** | 0.04* |
| **Bmac0156** (ch.7H) | 0.00 | 0.00 | 0.02** | 0.00 | 0.04*** | 0.01 |

**P*<0.05; ***P*<0.01; ****P*<0.001.
