# Supplementary material for: Adaptation and diversity along an altitudinal gradient in Ethiopian barley (Hordeum vulgare L.) landraces revealed by molecular analysis
Source: BMC Plant Biol. 2010 Jun 21;10:121. doi: 10.1186/1471-2229-10-121 (PMC3095281; doi:10.1186/1471-2229-10-121)
Supplement: Additional file 3 — Non-parametric correlation (Spearman's rho) between TESS and STRUCTURE clusters. [file 1471-2229-10-121-S3.DOC]

**Additional file 3.** Non-parametric correlation (Spearman’s rho) between TESS and STRUCTURE clusters.

| **STRUCTURE clusters** | **TESS clusters** | **Spearman’s *ρ*** | **P** |
| --- | --- | --- | --- |
| S1 | T1 | 0.72 | <0.0001 |
|  | T2 | 0.30 | <0.0001 |
| S2 | T5 | 0.45 | <0.0001 |
