# Supplementary material for: Adaptation and diversity along an altitudinal gradient in Ethiopian barley (Hordeum vulgare L.) landraces revealed by molecular analysis
Source: BMC Plant Biol. 2010 Jun 21;10:121. doi: 10.1186/1471-2229-10-121 (PMC3095281; doi:10.1186/1471-2229-10-121)
Supplement: Additional file 4 — Step-wise multiple regression analysis for STRUCTURE and TESS. The model was performed considering the STRUCTURE clusters as dependent variables and the TESS clusters as independent variables, from the data illustrated in Figures 4 and 5. [file 1471-2229-10-121-S4.DOC]

**Additional file 4.** Step-wise multiple regression analysis for STRUCTURE and TESS. The model was performed considering the STRUCTURE clusters as dependent variables and the TESS clusters as independent variables, from the data illustrated in Figures 4 and 5.

| **Dependant variable: STRUCTURE cluster S1 (green)** | | | **R^2^_adjusted_ = 0.84** |
| --- | --- | --- | --- |
| **Independant variables (TESS clusters)** | **SSQ** | **F-ratio** | **Prob(F)** |
| T1 | 23.96 | 735.04 | 0.00 |
| T2 | 20.32 | 623.36 | 0.00 |
| T4 | 8.29 | 254.51 | 0.00 |
| T3 | 3.28 | 100.66 | 0.00 |
| **Dependant variable: STRUCTURE cluster S2 (red)** | | | ***R^2^_adjusted_ = 0.84*** |
| **Independant variables (TESS clusters)** | **SSQ** | **F-ratio** | **Prob(F)** |
| T6 | 27.83 | 858.66 | 0.00 |
| T5 | 14.15 | 436.51 | 0.00 |
| T4 | 1.31 | 40.36 | 0.00 |
