# Supplementary material for: Adaptation and diversity along an altitudinal gradient in Ethiopian barley (Hordeum vulgare L.) landraces revealed by molecular analysis
Source: BMC Plant Biol. 2010 Jun 21;10:121. doi: 10.1186/1471-2229-10-121 (PMC3095281; doi:10.1186/1471-2229-10-121)
Supplement: Additional file 5 — Relationships between the molecular data and altitude. [file 1471-2229-10-121-S5.DOC]

**Additional file 5** Relationships between the molecular data and altitude^1^.

| **Locus** | **Chromosome** | **n. alleles per locus** | **R^2^** | **P** |
| --- | --- | --- | --- | --- |
| **HVM20** | 1 | 4 | 0.13 | 2.8E-05 |
| **Bmac0134** | 2 | 11 | 0.46 | 1.0E-15 |
| **Bmag0013** | 3 | 6 | 0.13 | 2.1E-15 |
| **HVM67** | 4 | 4 | 0.30 | 1.0E-12 |
| **Bmac0113** | 5 | 7 | 0.10 | 0.004 |
| **Bmac0040** | 6 | 11 | 0.47 | 7.0E-15 |
| **Bmac0156** | 7 | 23 | 0.37 | 5.4E-07 |

^1^ As analysis of variance (ANOVA) with the Wilcoxon non-parametric test.
