# Supplementary material for: Adaptation and diversity along an altitudinal gradient in Ethiopian barley (Hordeum vulgare L.) landraces revealed by molecular analysis
Source: BMC Plant Biol. 2010 Jun 21;10:121. doi: 10.1186/1471-2229-10-121 (PMC3095281; doi:10.1186/1471-2229-10-121)
Supplement: Additional file 6 — Types and frequencies of each of the seven qualitative traits, computed considering the genotypes that were assigned to one of the TESS clusters with a coefficient of membership (q) higher than 0.70 (from the data illustrated in Figure 5). [file 1471-2229-10-121-S6.DOC]

**Additional file 6** Types and frequencies of each of the seven qualitative traits, computed considering the genotypes that were assigned to one of the TESS clusters with a coefficient of membership (*q*) higher than 0.70 (from the data illustrated in Figure 5).

|  |  | **T1** | **T2** | **T4** | **T5** | **T6** |
| --- | --- | --- | --- | --- | --- | --- |
| **N° genotypes (*q* > 0.70)** | | **23** | **21** | **13** | **16** | **31** |
| **Trait** | **Status** | **Frequency (%)** | | | | |
| **Row number** | Two-rowed | 4.4 | 0.0 | 0.0 | 0.0 | 0.0 |
|  | Two-rowed deficient | 8.7 | 4.8 | 7.7 | 0.0 | 0.0 |
|  | Irregular | 21.7 | 80.9 | 76.9 | 50.0 | 74.2 |
|  | Six rowed | 65.2 | 14.3 | 15.4 | 50.0 | 25.8 |
| **Spike density** | Lax | 8.7 | 66.7 | 53.8 | 37.5 | 41.9 |
|  | Intermediate | 43.5 | 28.6 | 46.2 | 56.3 | 54.9 |
|  | Dense | 47.8 | 4.7 | 0.0 | 6.2 | 3.2 |
| **Lemma awn barbs** | Intermediate | 0.0 | 0.0 | 0.0 | 6.2 | 12.9 |
|  | Rough | 100.0 | 100.0 | 100.0 | 93.8 | 87.1 |
| **Glume colour** | White | 100.0 | 100.0 | 100.0 | 81.3 | 58.1 |
|  | Brown | 0.0 | 0.0 | 0.0 | 12.5 | 35.5 |
|  | Black | 0.0 | 0.0 | 0.0 | 6.3 | 6.4 |
| **Lemma type** | No lemma teeth | 13.0 | 47.6 | 46.2 | 68.8 | 48.4 |
|  | Lemma teeth | 87.0 | 52.4 | 53.8 | 25.0 | 51.6 |
|  | Lemma hair | 0.0 | 0.0 | 0.0 | 6.2 | 0.0 |
| **Length of rachilla hair** | Short | 52.2 | 90.5 | 92.3 | 100.0 | 100.0 |
|  | Long | 47.8 | 9.5 | 7.7 | 0.0 | 0.0 |
| **Lemma colour** | White | 82.6 | 52.4 | 38.5 | 50.0 | 16.2 |
|  | Tan/red | 4.4 | 9.5 | 38.5 | 12.5 | 3.2 |
|  | Purple | 4.3 | 4.7 | 0.0 | 0.0 | 3.2 |
|  | Black/grey | 8.7 | 28.6 | 23.0 | 37.5 | 74.2 |
|  | Yellow | 0.0 | 4.8 | 0.0 | 0.0 | 0.0 |
|  | Blue | 0.0 | 0.0 | 0.0 | 0.0 | 3.2 |
