# Supplementary material for: Adaptation and diversity along an altitudinal gradient in Ethiopian barley (Hordeum vulgare L.) landraces revealed by molecular analysis
Source: BMC Plant Biol. 2010 Jun 21;10:121. doi: 10.1186/1471-2229-10-121 (PMC3095281; doi:10.1186/1471-2229-10-121)
Supplement: Additional file 7 — Non-parametric correlation (Spearman's rho) between the TESS clusters for their morphological traits (from the data illustrated in Figure 5). [file 1471-2229-10-121-S7.DOC]

**Additional file 7** Non parametric correlation (Spearman’s rho) between the TESS clusters for their morphological (from the data illustrated in Figure 5).

| **TESS groups** | | **Spearman’s *ρ*** | **P** |
| --- | --- | --- | --- |
| T1 | T6 | 0.55 | 0.0068 |
| T1 | T5 | 0.65 | 0.0007 |
| T2 | T6 | 0.75 | <0.0001 |
| T1 | T4 | 0.77 | <0.0001 |
| T6 | T4 | 0.78 | <0.0001 |
| T5 | T2 | 0.80 | <0.0001 |
| T2 | T1 | 0.83 | <0.0001 |
| T5 | T4 | 0.83 | <0.0001 |
| T6 | T5 | 0.89 | <0.0001 |
| T4 | T2 | 0.96 | <0.0001 |
