# Supplementary figures and images for: Adaptation and diversity along an altitudinal gradient in Ethiopian barley (Hordeum vulgare L.) landraces revealed by molecular analysis
Source: BMC Plant Biol. 2010 Jun 21;10:121. doi: 10.1186/1471-2229-10-121 (PMC3095281; doi:10.1186/1471-2229-10-121)

## Genetic distance vs geographical distance

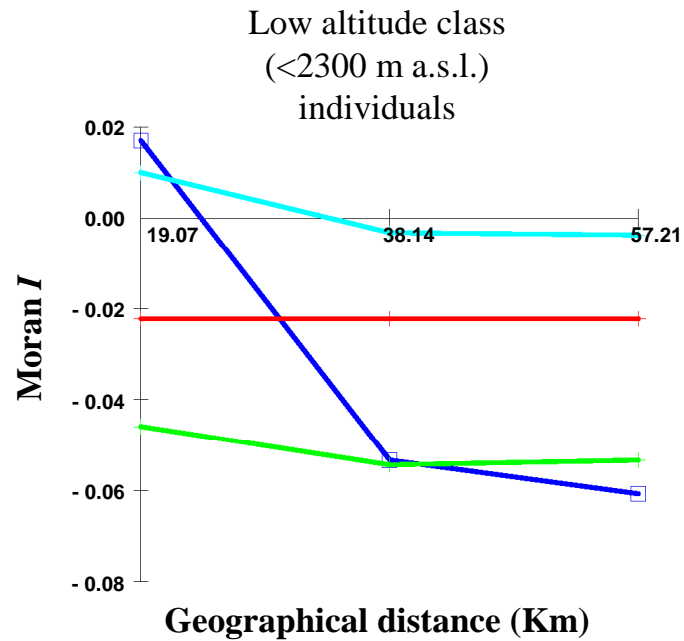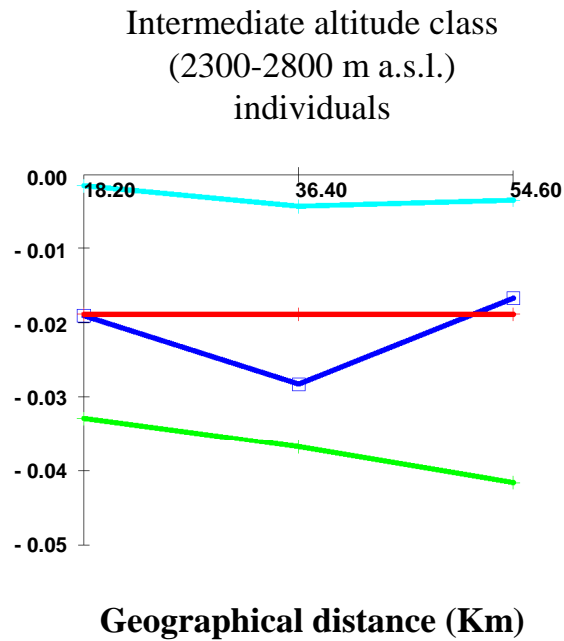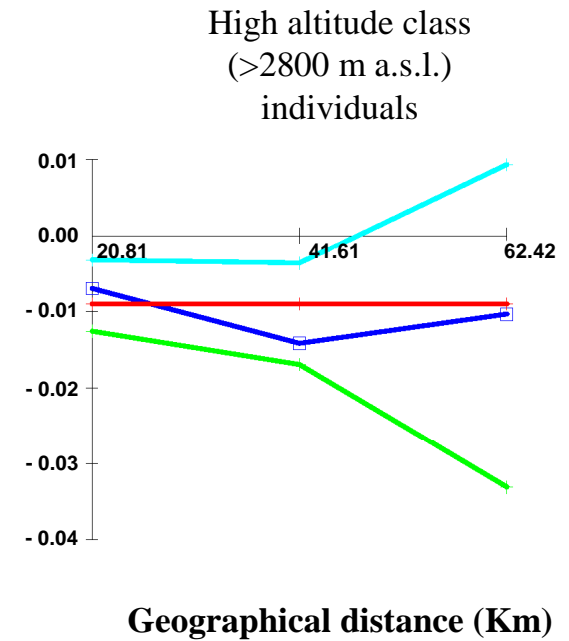

Supplement: Additional file 8 — Spatial autocorrelation analysis between the geographical and genetic distances, performed separately for the three altitude classes. See legend to Figure 7 for colour key. [file 1471-2229-10-121-S8.PDF]

**Additional file 9** Collection site coordinates of the barley landraces analysed.


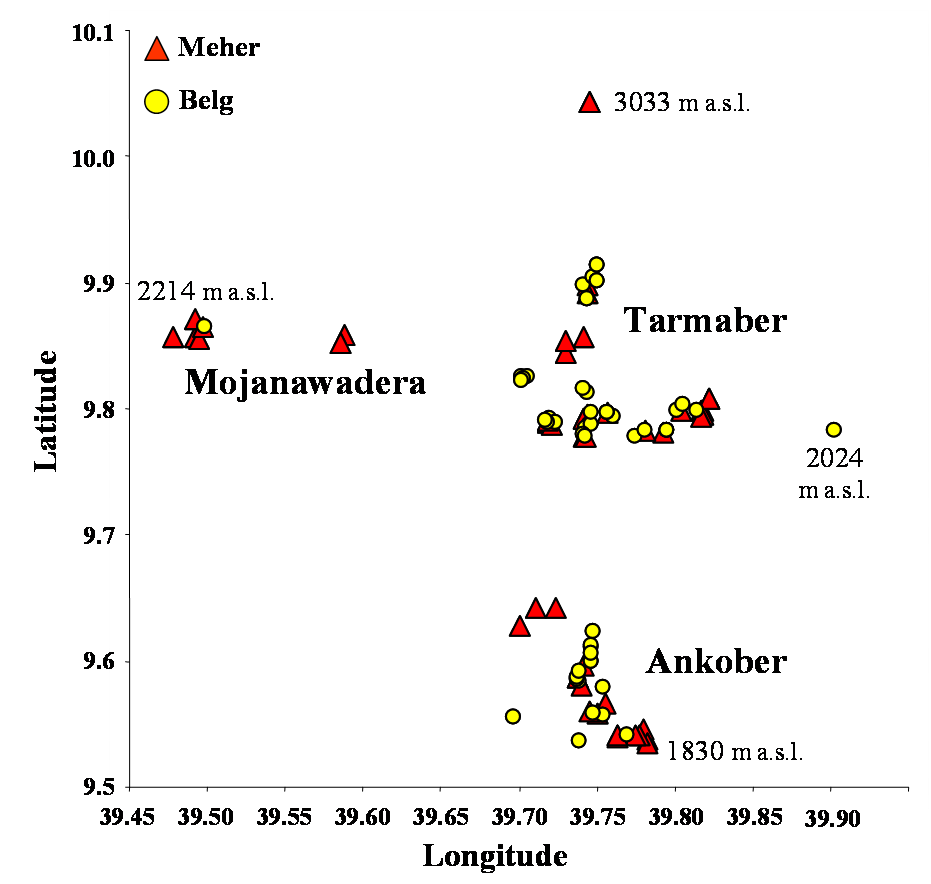

Supplement: Additional file 9 — Collection site coordinates of the barley landraces analysed. [file 1471-2229-10-121-S9.DOC]
