# Supplementary material for: Adaptation and diversity along an altitudinal gradient in Ethiopian barley (Hordeum vulgare L.) landraces revealed by molecular analysis
Source: BMC Plant Biol. 2010 Jun 21;10:121. doi: 10.1186/1471-2229-10-121 (PMC3095281; doi:10.1186/1471-2229-10-121)
Supplement: Additional file 10 — List of SSRs used in the present study. [file 1471-2229-10-121-S10.doc]

**Additional file 10** List of SSRs used in the present study.

| ***Chromosome*** | ***Locus*** | ***Repeat*** | ***Forward*** | ***Annealing temperature***  ***(°C)*** |
| --- | --- | --- | --- | --- |
| **1** | **HVM20** | GA_19_ | **Fw -** CTCCACGAATCTCTGCACAA  **Rev** - CACCGCCTCCTCTTTCAC | 57 |
| **2** | **Bmac0134** | AC_28_ | **Fw -** CCAACTGAGTCGATCTCG  **Rev -** CTTCGTTGCTTCTCTACCTT | 52 |
| **3** | **Bmag0013** | CT_21_ | **Fw -** AAGGGGAATCAAAATGGGAG  **Rev** - TCGAATAGGTCTCCGAAGAAA | 54 |
| **4** | **HVM67** | GA_11_ | **Fw -** GTCGGGCTCCATTGCTCT  **Rev -** CCGGTACCCAGTGACGAC | 57 |
| **5** | **Bmac0113** | AT_7_AC_18_ | **Fw -** TCAAAAGCCGGTCTAATGCT  **Rev -** GTGCAAAGAAAATGCACAGATAG | 52 |
| **6** | **Bmac0040** | AC_20_ | **Fw -** AGCCCGATCAGATTTACG  **Rev -** TTCTCCCTTTGGTCCTTG | 52 |
| **7** | **Bmac0156** | AC_22_AT_5_ | **Fw -** AACCGAATGTATTCCTCTGTA  **Rev -** GCCAAACAACTATCGTGTAC | 53 |
| **Amplification conditions:** PCR analyses were performed in a total volume of 20 µl, which contained 20 ng genomic DNA template, 50 pmoles of each primer, 200 µM dNTP, 2 mM MgCl_2_, 1x *Taq* polymerase buffer, and 1 U *Taq* DNA polymerase (Promega, Madison, WI, USA). One of the two SSR primers was end-labelled with 6-FAM or HEX. Amplifications were carried out with a Perkin-Elmer 9700 Thermal Cycler (PE Applied Biosystems, Foster City, CA, USA), with an initial denaturation of 5 min at 94 °C, which was followed by 30 cycles of 45 s at 94 °C, 45 s at X°C, and 45 s at 72 °C, plus 35 min of final extension at 72 °C. The X°C refers to the annealing temperatures for each primer pair. | | | | |
